# Where do mothers take their children for pneumonia care? Findings from three Indian states

**DOI:** 10.1371/journal.pone.0214331

**Published:** 2019-04-15

**Authors:** Rani Mohanraj, Shuba Kumar, Sylvia Jayakumar, Monica Agarwal, Bhavna Dhingra, Visalakshi Jeyaseelan, Saradha Suresh

**Affiliations:** 1 Samarth, Chennai, Tamilnadu, India; 2 Statshub, Chennai, Tamilnadu, India; 3 Department of Community Medicine and Public Health, King George’s Medical University, Lucknow, Uttar Pradesh, India; 4 Department of Pediatrics, All India Institute of Medical Sciences, Bhopal, Madhya Pradesh, India; 5 Department of Biostatistics, Christian Medical College, Vellore, Tamilnadu, India; ESIC Medical College & PGIMSR, INDIA

## Abstract

Childhood pneumonia accounts for 17% of IMR in India, posing a major health burden. With cultural beliefs influencing care seeking behaviour and disparities existing in health infrastructure across the country, an understanding of the underlying issues merits exploration. Study assessed prevalence of probable pneumonia and examined care seeking behaviour of mothers in three states, Madhya Pradesh (MP), Uttar Pradesh (UP) and Tamil Nadu (TN). This mixed methods study involved a household survey and qualitative interviews with mothers in three districts from each state. Households with children aged 2–59 months were screened to identify those with probable pneumonia; sub-sample of mothers participated in qualitative interviews. Care seeking behaviour was explored in the context of recognition of symptoms, nature of first care provided, time when care was sought outside the home and choice of health provider. Overall 17,442 children from 13,544 households were screened, of which 729 (MP), 752 (UP) and 713 (TN) children respectively, were identified with probable pneumonia; 72 mothers participated in the qualitative interviews. Three months period prevalence was estimated in study districts at 22.2%—MP 13.3%—UP and 8.4%—TN. Most mothers in MP and UP were not perceptive to severity of illness; type of care sought was often inappropriate, delayed, with home remedies and visits to unqualified care providers being their first response. In contrast, in TN, use of home remedies was minimal, going to untrained care providers, non-existent and more than 90% mothers sought appropriate care. Private doctors were the preferred choice among all mothers but utilization of government care was highest in TN (20%). Community health workers were underutilized, with less than 10% mothers consulting them. Need for educating mothers about appropriate care seeking and development of good health infrastructure as essential to attainment of better child health indices are advocated.

## Introduction

India has consistently been having the highest number of pneumonia and diarrhea deaths in children <5 years of age since 2000[1]and childhood pneumonia contributes to 17.1% of IMR (IMR) [2]. In response to the challenge of childhood illnesses, the Government of India’s National Policy on Children[3] and the Integrated Child Development Services (ICDS) have focused on survival, health and nutrition of mother and child which has contributed to the development of a more comprehensive child care strategy. This led to the setting up of a large number of health care centers, availability of medicines and establishment of tertiary care centers including transportation facilities in many states in India. These efforts contributed to a decline in under five mortality (U5MR)from 74 per 1000 live births in 2005–06 [4]to 50 in 2015–16 [5], still substantially short of the target laid down by the Millennium Development Goals (MDG IV) of 42 in 2015[6]. The challenge continues as the Sustainable Development Goals (SDG) has the new target of 25 per 1000 live births by the year 2030[7].

The Global Action Plan for Pneumonia and Diarrhea (GAPPD) score of India for pneumonia (measures the use of interventions that protect against, prevent and treat pneumonia and diarrhea)increased from 32% in 2014 to 41% in 2016because of continuous efforts in the roll out of Hib vaccine (pentavalent) and improvement in exclusive breastfeeding practices[8]. One of the observations of the GAPPD was that the rate of care seeking for symptoms of pneumonia increased by only 9 percentage points in South Asian countries,–from 62% in 2000 to 71% in 2015 and disparities existed between the richest and poorest households indicating country specific barriers[1]. Farooqui’s[9] modeling estimates on severe pneumonia, pneumococcal pneumonia and pneumonia deaths among the 29 Indian states showed many disparities. These estimates revealed that some states had a higher incidence of pneumonia and pneumonia deaths as compared to the national average. In the year 2010, the incidence of pneumonia in the northern state of Uttar Pradesh and the central state of Madhya Pradesh was 44.3 (CI: 40.7–48.3) and 41.4 (CI: 38–45.2), respectively which was higher than the national incidence rate of 30.7 (CI:28.1–33.5.) The study also reported that southern states like Tamilnadu (13.8; CI: 12.7–15.1), Kerala (11.5; CI: 10.5–12.5) and Karnataka (19.5; CI: 17.8–21.2) had lesser incidence of pneumonia and pneumonia deaths. These differences merit understanding and require an exploration of their underlying causes.

A systematic review of acute respiratory infection and pneumonia in children showed that one third mothers do not seek care for ARI for their children <5 years of age[10]. Gelsetzer’s [11] systematic review found issues like geography, cost of care, perception of the severity of illness and gender influenced care seeking behaviour. Poor health care systems and non availability of medicines in government health facilities were other barriers to care seeking for childhood pneumonia[12,13]. Very few studies from India have explored care seeking behavior for childhood pneumonia. Agarwal and Bajpai [14] found that only 40% of mothers’ perceived fast breathing and difficulty in breathing, the cardinal signs of pneumonia, as indicators of the disease. Other studies found inadequate utilization of public health services influenced by cultural beliefs and traditional family practices as detrimental to care seeking[15,16]. Studies carried out in sub-Saharan African countries and in the districts of Uganda showed that care seeking patterns were varied and were influenced by local cultural practices, community perceptions and availability of health infrastructure[17,18].

The limited studies on care seeking behaviour and the diversity in the incidence of pneumonia among the states encouraged us to undertake this study. Our study aimed to estimate recent episodes of pneumonia and also understand care seeking behaviour of mothers for their children aged 2–59 months in two high prevalence states, Madhya Pradesh (MP) in central India, Uttar Pradesh (UP) in north India and from a low prevalence state, Tamil Nadu (TN) in south India.

## Materials and methods

### Design and study sites

This mixed methods study involved a cross sectional household survey using a structured questionnaire and qualitative methods of semi-structured interviews (SSIs). The three Indian states of MP, UP and TN were selected based on the IMR published in the Sample Registration Survey (SRS-2015)[19]. The inclusion of UP and MP, were based on the evidence of achievement gaps on child health indicators as per MDG4 (i.e high IMR). The southern state of TN which served as a comparison site was chosen on account of its lower IMR. Three districts from each of these states were selected in consultation with the health departments of the respective state governments (Table 1).

**Table 1:** Selected states/districts and their IMR.

State/District	IMR
**Tamil Nadu State**	19*
Erode	16.8**
Tirunelveli	23.9**
Krishnagiri	23.9**
**Madhya Pradesh State**	50*
Bhopal	48***
Satna	83***
Panna	85***
**Uttar Pradesh State**	46*
Kanpur Nagar	37***
Faizabad	88***
Shravasti	96***

Source:

*Sample Registration Survey (SRS) 2015[19];

**Vital events Survey (2008)[20];

***Annual Health Survey 2012–13[21]

### Sample size, sample and sampling method

Sample size per state was calculated based on Mathew et al.’s study[10], according to which one third of caregivers in the community did not seek care for treatment of ARI for their children < 5 years. Assuming that 40% who do not seek care (or 60% who seek care), building in a margin of error of 5%, 95% confidence limits, and with a design effect of 2, the required sample size per state was 740 children with probable pneumonia in each state.

The sampling unit was the Health Sub-Centre (HSC) staffed by a Village Health Nurse (VHN)/ Auxiliary Nurse Midwife (ANM) and usually caters to a population of 5000–7000 with about 400 children< 5 years of age. The HSC constituted a cluster. A list of all the HSCs functioning in the selected districts was obtained and 30 HSCs per district were selected using the Population Proportionate to Size method. A central point in the selected HSC (cluster), like a panchayat office or a temple served as the starting point. From here, the study team turned to the right, commenced the house hold survey and continued until the sample size of 8 children with probable pneumonia was achieved.The District Level Household Survey (DLHS-4)[22] from TN (low prevalence State) had reported that 5.3% of children <5 years had suffered from ARI in the last two weeks prior to the interview. Further, our understanding of out-patient and in-patient data from a large hospital in Tamil Nadu shows that only about 10% or less of children with ARI are diagnosed to have pneumonia (personal communication: Dr. Suresh).We therefore increased our time frame to the last 3 months in order to obtain the desired sample size of 240 children per district.

Semi structured interviews were carried out on a sub sample of mothers whose child had symptoms of probable pneumonia. The sample included mothers i) who had sought treatment for probable pneumonia ii) who did not seek treatment for probable pneumonia and iii) whose child died due to pneumonia. We aimed to conduct interviews with a minimum of 3 mothers from each category. The District Health Officer (DHO) of the study districts was requested to provide documentation on childhood deaths that had occurred during the last 3 months. Their support was also sought to identify and locate mothers of such children. These mothers were then consented to participate in the SSIs.

#### Definitions

Our aim was to identify probable pneumonia and explore care seeking behaviour in the context of recognition of symptoms, nature of first care provided, time when care was sought outside the home, choice of health provider and mothers’ perceptions about their care seeking behaviour.

Index child: A child who had fast breathing with or without fever/cough/chest wall in-drawing/stridor in the last three months as part of a respiratory illness was defined as a case of probable pneumonia[23].

Appropriate Care Seeking: This referred to seeking care from a qualified allopathic provider be they government or private as against going to an unqualified care provider (UCP). Such providers have no formal medical training and dispense both allopathic and herbal medicines.

Early Care Seeking: This was defined a**s** seeking care within 24 hours of presentation of the symptom of probable pneumonia.

Care Seeking Behaviour: We explored care seeking behaviour from the perspectives of recognition of symptoms; nature of first care provided and type of care sought for treatment of the recent episode of probable pneumonia.

### Instruments

#### Household survey

A structured questionnaire was developed for the survey (S1 Text). An episode of fever, cold, fast breathing, chest wall in-drawing or stridor in the child during the last three months was elicited. Information with regard to use of home remedies, when care was sought outside home and where treatment was sought were also obtained. The questionnaire was translated into the local language of the study sites (Hindi and Tamil) and then pre-tested on a sample of 50 mothers -representative of our target population -per state before finalization. Care was taken to use the local terminology for fast breathing, chest wall in-drawing and stridor.

#### SSI guide

A guide was developed to explore care seeking behavior from the different categories of mothers. The interviews aimed to explore their perceptions about symptoms of pneumonia, practice of giving home remedies, when and from whom they sought care, and reasons for their choice of care provider.

### Data collection

Trained field investigators visited households in the selected HSC clusters and collected data using hand held electronic tablets in which the questionnaire was pre-loaded. A two step approach was adopted to identify an index child. In the first step, two screening questions “*Did the child have fever or cough in the last 3 months*?” and *“did the child have fast breathing/chest wall in drawing/stridor/grunt”* were asked. If the mother answered in the affirmative to either of the questions, as a second step, to confirm a child with probable pneumonia, mothers were asked if the child had fast breathing, chest wall in-drawing, stridor and grunt independently of the other symptoms. Those children who were reported to have had symptoms of fast breathing with or without other symptoms during the last three months were identified as having probable pneumonia and constituted the index child. Information on the most recent episode of probable pneumonia alone was considered. Additional information on care seeking practices was then elicited from this mother. In cases where two children from the same mother presented with symptoms of probable pneumonia, details of both children were recorded.

The SSIs with mothers were conducted in the local language by the field team who were trained by SK and RM. Audio recordings of interviews were transcribed and translated into English by persons proficient in the local language as well as English. Data collection was carried out from April 2016-January 2017 in all the three states.

### Analysis

#### Qualitative

All interview transcripts were coded using NVivo 8, qualitative analysis software (QSR international). Two coders (RM and SK) independently coded a few interviews and compared the coding framework to resolve differences in coding as a step to enhance validity. A thematic framework approach was adopted which involved multiple readings of transcripts to become familiar with the content, coding to help identify patterns, selecting quotes and finally, identifying emergent themes leading to interpretation of the data[24].

#### Quantitative

Data was entered, checked and analysed using SPSS software version 16.0 (SPSS Inc., Chicago, IL, USA). We calculated frequencies and percentages to estimate probable pneumonia for each of the districts and the state. Descriptive statistics were used to describe the characteristics of the sample. For continuous variables, mean and standard errors (SEs) were calculated while for categorical variables, frequencies and percentages were calculated. For skewed variables, the median was used instead of the mean. We then calculated cross tabulations and performed chi-square tests to identify demographic variables associated with the dependent variables i) ‘early care seeking’ and ii) ‘appropriate’ provider’.

### Ethics statement

Ethical approval was obtained from the Institutional Ethical Committee (IEC) of Samarth, and from those of King George’s Medical University (KGMU), Lucknow, UP and the All India Institute of Medical Sciences (AIIMS) Bhopal, MP. Semi–structured interviews were carried out in the privacy of the homes of the mothers at a time that was convenient to them. Their consent was obtained before doing the interviews. In cases where the mothers were non-literate, the consent form was read out to them in the presence of a witness. Consent was then obtained by getting the mother to either sign the form or place her thumb impression.

## Results

### Sample description

Qualitative Interviews: Table 2 presents the number of mothers who participated in the qualitative interviews from each of the stakeholder categories. In TN, we were unable to recruit mothers who had not sought care for treatment of probable pneumonia as they were either unavailable or refused participation.

**Table 2:** Number of qualitative interviews by stakeholder groups.

State	Sought Care	Not Sought Care	Child Died	Total
**MP**				
Bhopal	4	4	5	13
Panna	3	4	2	9
Satna	5	2	2	9
**Total**	**12**	**10**	**7**	**29**
**UP**				
Faizabad	4	3	2	9
Kanpur Nagar	3	-	1	4
Shravasti	4	1	3	8
**Total**	**11**	**4**	**6**	**21**
**TN**				
Erode	6	--	--	6
Krishnagiri	7	-	3	10
Tirunelveli	4	-	2	6
**Total**	17		5	22

### Household survey

A total of 13, 544 households from the three states of MP, UP and TN with children in the age range of 2–59 months were screened for the presence of symptoms of probable pneumonia. A total of 2047 mothers with 2194 index children in the 3 selected districts of MP, UP and TN were identified. The number of mothers’ with an index child was 595 in MP (mean age: 25.8-sd 3.8), 752 in UP (mean age 27.8- sd 3.9) and 700 in TN (mean age: 26.2- sd 4.6). The total number of mothers with an index child was less in MP, because in many households mothers reported more than one child with fast breathing in the last three months. There was a notable difference in education levels of mothers across the three states, with UP having the highest percentage of non-literate mothers (45%) as compared to MP (18%) and TN (7.3%) Mothers were predominantly home makers in all the three states. With respect to higher order birth, 45% of mothers in UP had more than 3 children, while it was 36% and 17% in MP and TN respectively. The mothers’ were predominantly from middle, lower, and lower middle socio economic strata which were assessed using a proxy indicator, namely dwelling type. Thus, families living in houses made of brick and concrete, termed as *‘pucca’* were categorized as middle income households. Those living in houses made of thatch and mud, termed as *‘kutcha’* were categorized as lower income households and those living in a combination of brick and thatch houses were categorized as lower-middle income households.

Information on care seeking behavior for 729 index children in MP, 752 from UP and 713 from TN was documented in this study. The index children in MP (mean age: 19 months; sd 12.9) were younger when compared to children from UP (mean age: 28.4 months; sd 15.4) and TN (mean age: 33 months; sd 15.4). The sample of index children in UP and TN were predominantly male while in MP there was a fairly equal distribution of boys and girls.

### Period prevalence of probable pneumonia

The 3 months prevalence of probable pneumonia was 22.2% in MP,13.3% in UP and 8.4% in TN. The districts of Satna in MP (27.8%), Shravasti in UP (13.8%) and Tirunelveli in TN (9.9%) showed higher prevalence of probable pneumonia as compared to the other districts (Table 3).

**Table 3:** Period prevalence of probable pneumonia.

Districts	Number of Households screened	Total no of children screened (>= 2 months to < = 59 months)	Number of children with probable pneumonia	Three months period prevalence of probable pneumonia
Bhopal	1067	1382	247	17.9
Panna	729	1014	237	23.4
Satna	634	880	245	27.8
**MP**	**2430**	**3276**	**729**	**22.2**
Faizabad	1405	1844	231	12.5
Shrawasti	1396	1940	267	13.8
Kanpur	1369	1859	254	13.7
**UP**	**4170**	**5643**	**752**	**13.3**
Erode	1850	2215	149	6.7
Tirunelveli	2575	3160	314	9.9
Krishnagiri	2519	3148	250	7.9
**TN**	**6944**	**8523**	**713**	**8.4**
**Total**	**13544**	**17442**	**2194**	**12.6**

### Care seeking behavior for probable pneumonia

Two themes were identified when exploring care seeking behavior among mothers from qualitative interviews and household survey. They were 1.Recognition of symptoms and nature of first care provided 2. Time when care was sought outside the home and reasons for the same. Results from MP and UP are presented first followed by findings from TN.

#### 1. Recognition of symptoms and nature of first care provided

In the qualitative interviews, mothers’ said that symptoms of fever, running nose, cough and difficulties in breathing indicated signs of illness in the child. According to many mothers *‘pasli-chalna’* (chest wall in-drawing), refusal or poor intake of food and lethargy in the child were indications of severity of the illness. Almost all mothers in MP and UP reported giving home remedies when they noticed symptoms of cold/fever/mild respiratory distress in the child for the recent episode of probable pneumonia. According to them giving home remedies is a tradition handed down from generations and they reposed immense faith in the effectiveness of these practices. They further said these home remedies came in handy as a first response, given that health facilities were sometimes located far away from their homes, there were difficulties in accessing them owing to poor transportation services, and they could not make decisions regarding care seeking in the in the absence of elder family member/husband. Mothers who had not sought care believed that the child’s symptoms were not serious enough to warrant any type of medical care and felt home remedies would suffice.

Only a few mothers in these states reported giving left over allopathic medicines. A couple of them had given medicines purchased from the local medical store by describing symptoms to the pharmacist. In many cases, home remedies were continued along with the left over allopathic medication. These home remedies differed widely from region to region. In TN, use of home remedies was not practiced except among a few mothers. Almost all mothers in TN gave left over allopathic medicines (mostly, paracetamol syrup) which was always available at home based on the advice of health care providers.

I got medicine from Medical…gave the medicine and *ghutti* (traditional herbal preparation). When the breathing was fast, mustard oil and kerosene oil was applied in small quantities… slowly got relief. *Ajwain* (carom seeds) and *hing* (Asfoetida), *haldi* (Turmeric). These 3 things were ground and given mixed with milk and I gave my child. (Not sought care 33234- Panna, MP).

He was coughing a lot and my father in law saw that there was *paslichalna* (chest in-drawing). The doctor nearby was telling that there was *pasli* (problem of chest in-drawing). We first went to take medicines and then we also did *JhaadPhoonk* (magico religious practice of chanting incantations and blowing holy ash over the child as a means of driving away evil spirits). I did oil massage, then there is the *barasingha (*a piece of deer horn that is finely ground) which was given by my mother in law, I rubbed it and applied it on the *pasli* (ribs). It is warm; hence the child had relief for some time. When he was having cough the entire night then we took him to the doctor to get medicines after which the child was relieved (Sought Care-160908-002, Faizabad-UP).

The results of the household survey also support much of these qualitative findings. Thus, (Table 4) administering home remedies was highly prevalent in UP and MP with more than half the sample ofmothers,66.6% and 50.5% respectively initiating home remedies for symptom management, as compared to only 4.5% of mothers in TN. Among the districts of the central and northern states, Panna in MP and Sharavasti in UP ranked highest with regard to use of home remedies at 72.2% and 79.8% respectively. Besides home remedies, mothers also reported giving leftover allopathic medicines and procuring medicines from the local pharmacy for symptom management (not mutually exclusive). The state of TN ranked the highest in use of leftover allopathic medicine (27.2%) followed by MP (14%) and UP (3.5%). Percentage of mothers who had contacted a CHW as a first response was very low in UP (0.3) and MP (2.2%) and marginally better in TN (8%).Avery small percentage of mothers also reported not giving any treatment to the child for the recent episode of probable pneumonia (MP = 5.6%; UP = 0.9; TN = 1.1%).

**Table 4:** Nature of first care provided.

District	Probable pneumonia n (%)	First care provided within 24 hours			
		Home Remedies n (%)	Left over medicine n (%)	From Medical shop n(%)	Consulted CHW
Bhopal	247	118 (47.8)	18 (7.3)	23 (9.3)	9(3.6)
Panna	237	171 (72.2)	51 (21.5)	22 (9.3)	2(0.8)
Satna	245	145 (59.2)	32 (13.1)	23 (9.4)	5(2.0)
**MP**	**729**	**434 (59.5)**	**101 (13.8)**	**68 (9.3)**	**16(2.2)**
Faizabad	231	140 (60.6)	7 (3.0)	15 (6.5)	--
Shrawasti	267	213 (79.8)	4 (1.5)	17 (6.4)	--
Kanpur Nagar	254	148 (58.3)	15 (5.9)	14 (5.5)	2(0.8)
**UP**	**752**	**501 (66.6)**	**26 (3.5)**	**46 (6.1)**	**2(0.3)**
Erode	149	5 (3.4)	10 (6.7)	2 (1.3)	3(2.0)
Tirunelveli	314	21 (6.7)	114 (36.3)	5 (1.6)	31(9.9)
Krishnagiri	250	6 (2.4)	70 (28.0)	9(3.6)	21(8.4)
**TN**	**713**	**32 (4.5)**	**194 (27.2)**	**16(2.2)**	**55(7.7)**
**Total**	**2194**	**967 (44.1)**	**321 (14.6)**	**130 (5.9)**	**73(3.3)**

#### 2. Time when first care was sought and reasons for choice of provider

When home remedies did not bring relief to the child, or when they noticed chest wall in-drawing, mothers sought treatment outside the home. Most mothers in MP and UP reported seeking care on the second or the third day. In MP, the choice of providers included the *jadibootiwala* (UCPs who dispensed herbal medicines), medicines procured from a medical shop or the private allopathic doctor. Most had sought care from a‘private doctor’ who gave ‘English medicines’ (allopathic). However, many mothers were unable to distinguish an unqualified provider from a qualified one. In the three districts of UP, mothers had predominantly sought care from UCPs who were easily accessible and affordable. They were referred to as *‘Jholachaap’*, as they travelled around the village with a *‘jhola’* (bag) carrying and dispensing both allopathic and herbal medicines. Mothers described that they were available, round the clock and also made home visits. While the majority of mothers never attributed financial barriers as impeding their choice of caregiver, most mothers from UP stated that treatment in government facilities was not completely free as was usually indicated. Medicines were not always provided and the cost of accessing these facilities added to the overall cost of treatment. In the districts of MP and UP, most mothers (except one) expressed faith in the practice of *JhaadPhoonk*. Such magico-religious practices continued even when the child was treated by other types of care providers. Except one mother from Panna district (MP) no one else reported contacting the CHW for advice on symptom management or care.

In contrast, none of the mothers in TN reported taking their child to a UCP. According to them though there were such kinds of care providers in their locality, they would never seek care for their child from them. Adults may go to them for treatment but children would not be taken. They also said that they had been strictly warned against seeking care from such individuals by their government HCPs. Many mothers reported contacting the CHWs over phone to seek her advice on treatment and next steps.

Not many mothers in MP and UP had utilized the government health facility. The reasons for this poor utilization were attributed to lack of trust and poor availability of medicines as a result they often had to buy medicines from private pharmacies. A few mothers who sought care from the government health facility expressed dissatisfaction with the care provided and with the uncaring and indifferent attitude of the staff. Doctors not being available in the facility and their own lack of clarity on clinic timings further added to their dissatisfaction. In TN, private allopathic providers were the predominant choice. Mothers who had sought care from both government and private care facilities, stated that while they were satisfied with the care provided in government facilities, the long waiting time and the crowds were a deterrent particularly when they felt that their child needed to be seen immediately.

When my daughter did not improve after treatment from local provider, he advised us to go to hospital in Jabalpur…. there was not much time as she was serious…so I admitted my daughter in government hospital in Rewa (Sanjay Gandhi Medical hospital). Problem in Government is that neither they care nor they listen to us. There were 3–4 patients on each bed, there was no place to sit and it was very dirty. So he (my brother in law) told us to go to private hospital in Nagpur (Child Died 33066- Satna, MP).

She had cold for a few days. We did not take her anywhere….we bought syrup from medical store. There was some improvement from that, then again it became the same. When the medicine finished then again the p*aanjar* (chest wall in-drawing) started. She recovered after having *paanjar* 2–4 times like this. Then she became very sick … We took her in the morning only. First we went to Gilaula government hospital and then the doctor told us to take child to Behraich district hospital as the child has pneumonia and he can’t treat her as there were no facilities to treat her. He scolded us and said that ‘child was ill for so long but you people didn’t bring her here. (Child died 161221-001-Shravasti, UP).

My daughter developed fever and cold in the evening and she had problems breathing… I took her to the private doctor nearby…I usually take her to the government hospital but since it was late evening I took her to private doctor…Doctors will be available in the morning only in government hospitals. The doctor gave nebulisation and at night there was no breathing problem. From next day I gave medicine and step by step she became alright. Doctor gave medicines for 3 days (Sought Care 13402- Tirunelveli, TN).

Quantitative result on time when first care was sought was categorized as early care seeking (within 24 hours of presentation of symptoms) and delayed care seeking (after 24 hours of presentation of symptoms). The median time was 2 days in TN and MP and 3 days in UP. Among the three states TN had the highest percentage of mothers who had taken the child within 24 hours (83%), followed by UP (70%) and MP (59%). Among the districts in these states, maximum percentage of delayed care seeking was seen in Panna,MP (51%), Shravasti, UP (37%) and Erode, TN(35%)(Table 5).

**Table 5:** Time when first care was sought.

	MP n(%)			UP n(%)			TN n(%)		
	Bhopal	Panna	Satna	Kanpur Nagar	Shravasti	Faizabad	Erode	Tirunelveli	Krishnagiri
<24 hours	172 (69.64)	116 (48.95)	140 (57.14)	186 (73.23)	169 (63.30)	175 (75.76)	97 (65.10)	262 (83.44)	234 (93.60)
>24 hours	75 (30.36)	121 (51.05)	105 (42.86)	68 (26.77)	98 (36.70)	56 (24.24)	52 (34.90)	52 (16.56)	16 (6.40)
**Total**	**247**	**237**	**245**	**254**	**267**	**231**	**149**	**314**	**250**

With regard to the type of provider sought, our findings revealed that the private allopathic doctor was the preferred choice reported by mothers in MP. More than 60% of mothers in the three districts had sought care from a private doctor and a small percentage (12%) had sought care in a government facility. In the districts of UP, care sought from UCPs was reported by a majority of mothers (71%) while in TN the majority of mothers (75%) had sought care from private allopathic doctors. At least 20% of the mothers had sought care from a government facility in Tirunelveli district (Table 6).

**Table 6:** Type of care provider sought.

Site	Type of Provider				
	Govt n(%)	Private n(%)	UCP n(%)	Missing n(%)	Total n(%)
Bhopal	26 (10.5)	172 (69.64)	39 (15.8)	10 (4.05)	**247**
Panna	28 (11.8)	181 (76.3)	16 (6.7)	12 (5.1)	**237**
Satna	35 (14.3)	188 (76.7)	2 (0.8)	20 (8.1)	**245**
**MP**	**89** **(12.2)**	**541 (74.2)**	**57** **(7.8)**	**42** **(5.8)**	**729**
Kanpur Nagar	19 (7.5)	69 (27.2)	160 (63)	6 (2.4)	**254**
Shravasti	23 (8.6)	13 (4.9)	212 (79.4)	19 (7.1)	**267**
Faizabad	11 (4.8)	45 (19.5)	161 (69.7)	14 (6.1)	**231**
**UP**	**53** **(7.0)**	**127 (16.9)**	**533 (70.9)**	**39** **(5.2)**	**752**
Erode	28 (18.8)	111 (74.5)	**-**	10 (6.7)	**149**
Tirunelveli	64 (20.4)	244 (77.7)	**-**	6 (1.9)	**314**
Krishnagiri	48 (19.2)	180 (72.0)	**-**	22 (8.8)	**250**
**TN**	**140** **(19.6)**	**535 (75.0)**	**-**	**38 (5.5)**	**713**

The result of the association between mother and child demographic characteristics to early care sought is presented in (Table 7). In MP, early care seeking was significantly associated with type of housing and gender of the child. Mothers who lived in ‘*pucca’* houses (p = 0.01) sought care within 24 hours and male children were taken to a care provider within 24 hours when compared to girl children (p = 0.02). In UP, education was significantly associated with early care with mothers with more years of schooling seeking care within 24 hours (p = 0.01).In TN, early treatment was positively associated with age of the child. Younger children were taken for care within 24 hours (p = 0.00).

**Table 7:** Association of socio-demographic characteristics to early care sought.

Early vs delayed care		MP			UP			TN		
		Within 24 hrs	After 24 hrs	P* value	Within 24 hrs	After 24 hrs	P* value	Within 24 hrs	After 24 hrs	P* value
**Mothers’ characteristics**		**N = 349 (%)**	**N = 246 (%)**		**N = 530 (%)**	**N = 222 (%)**		**N = 580 (%)**	**N = 120 (%)**	
Age in years	<25	102 (64.2)	57 (35.8)	0.26	108 (70.6)	45 (29.4)	0.82	215 (85.7)	36 (14.3)	0.17
	26–35	220 (56.7)	168 (43.3)		386 (70.8)	159 (29.2)		329 (80.6)	79 (19.4)	
	>35	27 (56.2)	21 (43.8)		36 (66.7)	18 (33.3)		36 (87.8)	5 (12.2)	
Education	Non-literate	56 (53.8)	48 (46.2)	0.18	222 (65.5)	117 (34.5)	0.01	40 (78.4)	11 (21.6)	0.08
	Upto 5th class	72 (57.1)	54 (42.9)		84 (69.4)	37 (30.6)		56 (82.4)	12 (17.6)	
	6- 10th class	179 (58.5)	127 (41.5)		140 (74.5)	48 (25.5)		317 (80.7)	76 (19.3)	
	> 10th class	42 (71.2)	17 (28.8)		84 (80.8)	20 (19.2)		167 (88.8)	21 (11.2)	
Number of Children	0–2	225 (59.2)	155 (40.8)	0.72	294 (71.4)	118 (28.6)	0.56	476 (82.1)	104 (17.9)	0.22
	3 or more	124 (57.7)	91 (42.3)		236 (69.4)	104 (30.6)		104 (86.7)	16 (13.3)	
Type of housing	Kutcha	215 (54.4)	180 (45.6)	0.01	153 (65.1)	82 (34.9)	0.05	364 (82.7)	76 (17.3)	0.31
	Pucca	71 (68.9)	32 (31.1)		174 (75.3)	57 (24.7)		205 (82.3)	44 (17.7)	
	Combined	63 (64.9)	34 (35.1)		203 (71.0)	83 (29.0)		11 (100.0)	-	
**Child Characteristics**		**N = 428 (%)**	**N = 301 (%)**		**N = 530 (%)**	**N = 222 (%)**		**N = 593 (%)**	**N = 120 (%)**	
Gender	Male	233 (62.6)	139 (37.4)	0.03	313 (69.2)	139 (30.8)	0.36	331 (83.0)	68 (17.0)	0.86
	Female	195 (54.6)	162 (45.4)		217 (72.3)	83 (27.7)		262 (83.4)	52 (16.6)	
Age	<12 months	86 (63.7)	49 (36.3)	0.06	205 (71.7)	81 (28.3)	0.68	81 (93.1)	6 (6.9)	<0.001
	13–36 months	217 (60.8)	140 (39.2)		261 (69.0)	117 (31.0)		270 (85.2)	47 (14.8)	
	>36 months	125 (52.7)	112 (47.3)		64 (72.7)	24 (27.3)		242 (78.3)	67 (21.7)	

* chi-square test

Table 8 presents the findings of demographic characteristics of mothers and their children and its association with appropriate care seeking behaviour. In MP, appropriate care was associated only with type of housing (p = 0.00).In UP, many demographic characteristics were significantly associated with appropriate care. Age of mother was significantly associated, with younger mothers seeking care from an appropriate provider (p = 0.01). Mothers with 2 or less number of children (p = 0.00), and living in ‘pucca’ houses sought care from an appropriate provider. With regard to child characteristics, it was noted that a greater proportion of male children were taken to an appropriate care provider as compared to female children (p = 0.001).

**Table 8:** Association of socio-demographic characteristics to appropriate care sought.

Appropriate vs Inappropriate Care		MP			UP		
		Qualified Provider	Unqualified Provider	P* value	Qualified Provider	Unqualified Provider	P* value
**Mothers’ characteristics**		**n = 512 (%)**	**n = 48 (%)**		**n = 180 (%)**	**n = 533 (%)**	
Age in years	<25	142 (93.4)	10 (6.6)	0.59	50 (34.0)	97 (66.0)	0.02
	26–35	330 (90.7)	34 (9.3)		116 (22.6)	397 (77.4)	
	>35	40 (90.9)	4 (9.1)		14 (26.4)	39 (73.6)	
Education	Non-literate	85 (94.4)	5 (5.6)	0.58	50 (16.0)	263 (84.0)	<0.001
	Upto 5th class	109 (89.3)	13 (10.7)		32 (27.6)	84 (72.4)	
	6- 10th class	266 (91.7)	24 (8.3)		52 (28.6)	130 (71.4)	
	> 10th class	52 (89.7)	6 (10.3)		46 (45.1)	56 (54.9)	
No. of Children	0–2	330 (91.7)	30 (8.3)	0.79	118 (29.8)	278 (70.2)	<0.001
	3 or more	182 (91.0)	18 (9.0)		62 (19.6)	255 (80.4)	
Type of housing	Kutcha	341 (92.7)	27 (7.3)	<0.001	50 (22.9)	168 (77.1)	0.02
	Pucca	81 (81.0)	19 (19.0)		71 (31.8)	152 (68.2)	
	Combined	90 (97.8)	2 (2.2)		59 (21.7)	213 (78.3)	
**Child Characteristics**		**n = 630 (%)**	**n = 57 (%)**		**n = 180 (%)**	**n = 533 (%)**	
Gender	Male	330 (93.5)	23 (6.5)	0.08	127 (29.5)	304 (70.5)	<0.001
	Female	300 (89.8)	34 (10.2)		53 (18.8)	229 (81.2)	
Age	<12 months	122 (93.1)	9 (6.9)	0.767	73 (26.6)	201 (73.4)	0.650
	13–36 months	306 (91.1)	30 (8.9)		84 (23.7)	270 (76.3)	
	>36 months	202 (91.8)	18 (8.2)		23 (27.1)	62 (72.9)	

* chi-square test

## Discussion

Our study explored care seeking behaviour of mothers across nine districts in three Indian states using a combination of qualitative and quantitative methods. The narratives of the mothers provided rich insights into the phenomena of care seeking while the quantitative data provided the empirical evidence and enhanced the validity of our findings.

### Recognition of illness and delayed care

Ability of the mother to recognize respiratory illness is the key to seeking care and reduction in mortality as many ARI deaths occur within 3–5 days of disease onset[25].The fact that more than 95% of mothers in our study had resorted to some kind of care for their child indicated that they were able to recognize illness. However, they were often unable to perceive severity of illness requiring urgent care resulting in delayed care seeking. Findings from our qualitative narratives revealed that fast breathing was not perceived as a danger sign. For many mothers in MP and UP, chest wall in-drawing was a more serious condition than fast breathing. This is corroborated by many studies both in India and other developing nations[15,26,27]. Poor understanding of severity of illness demand that the focus of community educational messages should stress more on recognition of early symptoms of cough with/without fever which may have more value than perhaps educating mothers about fast breathing viz-a-viz chest wall in drawing which focuses on severity of illness.

### Home remedies and health education

Our findings revealed that a large proportion of mothers (59% in MP and 66.5% in UP) had resorted to home remedies as a first response. Use of home remedies has been reported in many recent studies from Asian and African countries which are influenced by cultural practices, advice from elders at home, recovery on previous occasions of illness, monetary constraints and compromised decision making ability of the mothers[16,28–30]. Contrary to the wide spread practice of home remedies in these two states, in TN less than 5% of mothers resorted to home remedies. Mothers stated that during their ANC visits at the government/private facilities, they were advised against giving home remedies, were encouraged to practice exclusive breast feeding and seek care as soon as they noticed early signs of illness in their child. This finding is consistent with studies from Ethopia [31] and Nepal [32] where mothers who accessed a qualified care provider during their ANC visits showed better health care seeking and good health outcomes in their child, attributable to the health education received during these visits. One of the reasons for better child health indices (lower IMR and lower prevalence of probable pneumonia) in TN could be attributed to the exposure of mothers to these continued health messages (immunization, nutrition, childhood illnesses and danger signs) received during their ANC visits. According to the National Family Health Survey (NFHS-4)[5], the percentage of mothers who had at least 4 ANC visits in rural TN stands at 81% while it is only 29.6% and 21.7% in MP and UP respectively which gives fillip to the fact that consistent health education can lead to better health practices by mothers for their children. Governments in MP and UP should invest in efforts to improve ANC visits of mothers.

### Appropriate vs inappropriate care seeking and early vs delayed care seeking

In all the three states there were a good proportion of mothers who had sought care within 24 hours. As regards delayed care seeking, it was the highest in MP with 40% seeking care after 24 hours. In large part, the practice of home remedies which was considerably high at 59%, and which was the first response of most mothers in MP can be held responsible for delays in care seeking. In UP, despite 67% of mothers resorting to home remedies, a good proportion (70%) had sought care within 24 hours. However, care sought was mostly inappropriate, with appropriate care sought at a low of 24%. The low percentage of care seeking from appropriate care providers in UP was less than the average of 48% in sub-Saharan African countries[33]. In MP although mothers predominantly reported going to private care providers they were unable to confirm if the providers were qualified or unqualified. In rural India, UCPs who dispense allopathic medicine are very common. They have no medical training and would probably have assisted a doctor or worked in a pharmacy. The role played by UCPs has been well documented in earlier studies [34,35]. We confirmed the presence of a large number of UCPs in the study districts of MP and understood that mothers predominantly seek care for their children from them (Dhingra B, site principal investigator, personal communication). Apart from being easily accessible, available and affordable, people have great faith in UCPs and believe in their ability to heal [15]. The practice of seeking care from UCPs has also been reported in many African countries [36,37].The low level of trust, dissatisfaction with care provided and inadequate drug supplies in government facilities, non availability of qualified providers in 24/7 health facilities, indifferent attitudes of care provider have acted as major barriers to seeking care from public health facilities in UP and MP. These barriers have been reported in many studies in LMIC countries including India [15,36,38].The qualitative narratives of mothers from MP and UP in our study described the difficulties they faced in accessing government facilities owing to poor public transport. Some were unaware of the ambulance services while others complained that the ambulance would never arrive on time, issues pointed out in another study done in UP and Bihar [15]. It therefore falls on the governments of MP and UP to educate the public on the harms of seeking care from UCPs while simultaneously improving health infrastructure, increasing awareness about ambulance services and their purpose as a means to enhancing their better utilization.

Findings from TN showed that more than 90% had sought appropriate care either from private providers or at government facilities, with the majority seeking care from private providers. A little over 80% had taken their child to an appropriate care provider within 24 hours. Though the presence of UCPs was acknowledged by mothers, the fact that none of the mothers had sought care from them attests to the availability of a good health system and better awareness among mothers about the harms of seeking inappropriate care. Mothers were also aware of the ambulance services which were mostly reported to arrive on time. Although utilization of government hospitals was far higher in TN than in MP and UP, the long queues and the refusal of government doctors to administer injections to the sick child have acted as deterrents to their higher utilization. The general belief by most people in the community is that giving an injection would lead to fast recovery in the child.

We also found certain demographic characteristics of mother and child to be associated with early and appropriate care and state wise differences in these associations. While we did not document wealth quintile of the participants we can to some extent infer that socio economic status had an influence on early and appropriate care seeking in MP and UP confirming findings from studies carried out in India and other developing nations [10,28]. The practice of home remedies, cost incurred in reaching a health care facility or reaching an appropriate provider and treatment cost, influenced time when care was sought and also the type of provider from who care was sought. In MP, male children were taken early for care as compared to girl children, findings which are similar to trends reported from a study in Nigeria[39] In UP, only education level of mothers’ influenced early care seeking, similar to studies in India and other countries like Africa and Peru indicating that maternal education is an important contributor for improved child health [38,40]. In TN younger children were taken for care within 24 hours, an indication of good awareness about the risks involved in delaying care for younger children, given that incidence of pneumonia is higher in children < 2 years of age[18].

In UP appropriate care sought was influenced by many socio-demographic characteristics of the mother in resonance with other studies. Younger mothers sought appropriate care when compared to older mothers, similar to findings in other studies[41]. Education level of mothers also influenced appropriate care seeking [18,36].Studies from West Bengal and Uttar Pradesh showed that male children were taken for appropriate care while girl children were taken to unqualified care providers similar to the findings in our study [42]. The strong cultural practice of preferential treatment for males in MP and UP which is widely prevalent even today suggests the need to educate families against such discriminatory practices.

### Community health workers

Results of our survey also showed that less than 10% of mothers had consulted CHWs in all the states. Mothers were not aware of the roles of the CHWs in initiating care for childhood illnesses like pneumonia, in the districts of UP and MP. Previous studies also found that the ANM’s knowledge about danger signs of pneumonia was inadequate and the community had not consulted them for management of pneumonia in their child [16].Thus the CHWs, despite their fairly large presence have been found to be an under-utilized force. However, there are encouraging findings from studies on community based interventions and community case management (CCM) of pneumonia wherein CHWs were trained to detect and treat pneumonia with antibiotics. A systematic review of community interventions showed an increase in care seeking behavior for pneumonia by 13%and 32% reduction in pneumonia mortality when CHWs provided antibiotics for pneumonia management [43]. In Pakistan, sensitization meetings for opinion leaders in the community, awareness programmes and training of the Lady Health Workers over a period of time, resulted in an increase in care seeking behavior by 48% [44].Our qualitative findings revealed that the CHW is perceived more as health care facilitator rather than a health care provider. Even in TN, where 7.7% of mothers had consulted the CHW, it was more for advice rather than seeking care. Despite trained CHWs available and functioning in the study areas many mothers were either unaware of their roles as care providers or were reluctant to seek care from them. Furthermore, a consultation with the CHW was often over the phone as was seen in TN as these CHWs were not living in the same neighborhood. Given that CHWs have to cover populations that are spread over a large area with poor accessibility, the use of mobile phones as happening in TN, may be a viable alternative in MP and UP. Our desk review on the existing health systems in these three states showed a vast difference between them with respect to the number of health facilities and health care providers available. The district of Shravasti in UP, with the highest prevalence of pneumonia has only 12 Primary Health Centers (PHCs), 6, Community Health Centers (CHC) and one tertiary hospital. In Panna, the district with the highest prevalence in (MP), the number of PHCs was 14, CHCs was 6 and there was only one government hospital. The national standards for health facilities which advocates one PHC for 30–40,000 population was compromised in thesestates. On the contrary, in TN, the number of PHCs in the high prevalence district of Tirunelveli stood at 70, with 19 CHCs and 14 Government hospitals. There is therefore a need for political and administrative commitment by the governments in MP and UP to strengthen their health infrastructure by providing more 24/7 health facilities with trained health personnel and ambulances services for transport. There is an urgent need to address this gap if India needs to achieve the SDG goal in childhood pneumonia management.

One of the limitations of our study was that we relied on the self reports of mothers for determining presence of symptoms of probable pneumonia which may be subject to recall bias. One other limitation was with respect to identification of cause of death. In the absence of proper documentation of mortality data in UP and MP we had to rely on the self reports of the mothers and/or seek help from the CHWs to identify mothers whose child had died due to probable pneumonia. In TN, while we had access to the child mortality data from the respective district health offices (DHS), the cause of death was not always adequately documented.

## Conclusion

Our study documented recent episodes of probable pneumonia among children 2–59 months of age with specific focus on where they were taken for care by their mothers. Findings of our study reiterated results from low and middle income countries that early and appropriate care seeking are essential management strategies for treatment of childhood pneumonia. An equally important finding was that care seeking behaviour was largely driven by the way mothers perceived the illness, their own understanding of illness severity coupled with the value they saw in seeking a particular kind of care be it appropriate or inappropriate. Added to this were issues related to accessibility and availability of health care infrastructure. Thus in MP delays in seeking care were commonly seen while in UP seeking inappropriate care predominated. In contrast, in TN not only was care seeking both early and appropriate but there was a good degree of trust reposed in the government healthcare system that further influenced its usage by the mothers. If we have to draw lessons from the low prevalence state, then it is imperative that appropriate care be made available, accessible and efforts be taken to engender trust towards the government health care system in communities. In conclusion, two strategies for pneumonia management are recommended: a)educate and empowermothers to seek early and appropriate care for respiratory illness in their children b) Improve and monitor the delivery of health care services at the government health facilities. These steps would be key in enabling India march towards attainment of the SDG goal of less than 25 deaths per 1000 by 2025.

## Supporting information

S1 Table Database. (XLSX)

S2 Table Variable description. (XLSX)

S1 Text Survey questionnaire. (DOCX)
