# Supplementary material for: Where do mothers take their children for pneumonia care? Findings from three Indian states
Source: PLoS One. 2019 Apr 15;14(4):e0214331. doi: 10.1371/journal.pone.0214331 (PMC6464169; doi:10.1371/journal.pone.0214331)
Supplement: S1 Text — (DOCX) [file pone.0214331.s003.docx]

**SAMARTH**

**Care seeking in Childhood Pneumonia Management: An Exploratory Study**

**Quantitative Household Survey Data Collection Form-**

**Respondent : Mother of Child Under 5yrs of age**

Vanakkam. I am coming from Samarth, Chennai.

Namaste. I am coming from AIIMS, Bhopal.

Namaste. I am coming from KGMC, Lucknow.

I am doing a household survey of families with children under 5 years of age.Iwould be enquiring about whether your child had signs and symptoms of pneumonia in the last 3 months and what treatment was given to the child.

State : TN : 1

UP: 2

MP:3

District: Erode: 1

Krishnagiri: 2

Thirunelvelli: 3

Record ID : 001……

Data record number starts from 001

*(Unique ID includes State code , district code and Data record number. Eg 21001, 21002 etc)*

**For administrative purpose only**

| 1. Unique ID No(*will be filled in later*) | vi. Block |
| --- | --- |
| 1. Date of Interview | vii. HSC |
| 1. Interviewer Name | viii. Village |
| 1. State | ix. Address of the interviewee |
| 1. District |  |

**1.Demographic Data**

|  | **1. Mother** | **2.Father** |
| --- | --- | --- |
| **Name** | 1.1 | 2.1 |
| **Age** | 1.2 | 2.2 |
| **Education** | 1.3 | 2.3 |
| **Occupation** | 1.4 | 2.4 |
| **Religion** | 1.5 | 2.5 |
| **Community** | 1.6 | 2.6 |

**Education codes**: 1. Non literate 2. Primary school 3. Middle school 4.High school (9^th^ -10^th^ std) 5. Higher Secondary (11^th^ to 12^th^Std) 6. Above high school

**Occupation codes**: 1. Home maker 2.Daily labourer 3. Skilled worker (painter, carpenter, plumber etc) 4. Self employed 5. Others_________________________

**Religion codes:** 1. Hindu 2. Christian 3.Muslim 4. Others

**Community codes** 1. SC 2.ST 3. Others *(Specify if MBC, BC, OC, FC, etc.)*

1. What is the colour of your ration card?

1. Blue/Green/Red 2. Yellow 3. White 4. No card

1. Family Size (no of persons living in the housewho share a kitchen): ____________
2. Family type: 1. Nuclear *(Father, mother and children)* 2. Non- nuclear
3. Total no of children for the interviewee ________________
4. Type of house 1. Kuccha 2. Pucca 3. Both
5. No. of children under five years(**child must not have completed 5 years on date of interview**) in the household*:*

| S.No | 8.1 Name | 8.2 Date of Birth | 8.3 Gender  1. Male  2. Female | 8. 4 Did the child have fever or cough during the last 3 months  1. Yes 2. No | 8.5 If Yes did the child also have any one of the following symptoms - fast breathing/chest wall in drawing/stridor /grunt in the last 3 months?  1. Yes 2. No  3. Don’t remember | 8.6 Diagnosis  Pneumonia  1.Yes 2. No |
| --- | --- | --- | --- | --- | --- | --- |
| 1 |  |  |  |  |  |  |
| 2 |  |  |  |  |  |  |
| 3 |  |  |  |  |  |  |
| 4 |  |  |  |  |  |  |

*(Child with pneumonia is the index child. Circle the name of the index child in the box. If more than one child under 5 yrs with pneumonia is present in the household, use this current schedule for the youngest child and another schedule for the older child. If mother reports no child under 5 yrs of age with pneumonia in the last 3 monthscontinue with questionnaire and complete section5)*

*If mother has more than 1 child under 5 yrs of age, please record immunization status& nutrition practices of each child in the separate immunization/nutritionschedules provided below*

**2.Environmental Issues**

9. Does any family member living in this household smoke tobacco ***within the home***?

1. Yes 2.No

10. What type of fuel is used by you for cooking? *(Circle all that apply)*

1. LPG 1. Yes 2. No
2. Kerosene 1. Yes 2. No
3. Biofuel (dried dung, wood etc) 1. Yes 2. No

11. Note if cooking is done …..

1. Inside the home 2. Outside the home 3. Both

12. Note presence of open drainage outside or surrounding the home

1. Yes 2. No

13. Note/ask presence of factory emitting smoke/pollutants within 1 km of the home

1. Yes 2. No

14. Note if domestic animals *(these include cows, buffalo, goats, sheep, chicken, dogs, cats)*are :

1. Sheltered within the home 2.Housed in a separate space outside home

3. No domestic animals present

15. Note if Toilet facility is: *(circle all that apply)*

1. Within the house 2. Outside public toilet facility 3. Shared toilet within the compound 4.Open defecation in fields

**3. Hygiene Practices**

16. Do you clean your hands every time after you have used the toilet?

1.Always 2. Majority of the times 3.Sometime 4. Rarely

17. How do you usually clean your hands after you have used the toilet?

17.1 Rinse with water only 1. Yes 2. No

17.2 Wash with soap and water 1.Yes 2. No

17.3 Rub hands with sand 1. Yes 2. No

18. Before feeding child *(includes breast feeding and feeding by hand)* do you clean your hands?

1.Always 2. Majority of the times 3.Sometimes 4. Rarely

19. How do you usually clean your hands before feeding yourchild?

19.1 Wash with water only 1. Yes 2. No

19.2 Wash with soap and water 1. Yes 2. No

19.3 Wipe hands on cloth 1. Yes 2. No

20. Did any govt. health worker visit you**in your home** during the last month?

1. Yes 2. No 3. Don’t remember

21. Did any health worker (VHN, ASHA, AWW etc) talk to you regarding danger signs*(stridor, fast breathing, chest-wall in drawing and difficulty in breathing*) in children **during the last month?**

1. Yes 2. No 3. Don’t remember

21.a Specify who spoke with you regarding danger signs ? _________________________

21.1. Did any health worker talk to you about hygiene practices*(hand washing, not exposing the children to any person with infection, not allowing person to kiss the child)***during the last month?**

1. Yes 2. No 3. Don’t remember

**4. ImmunisationStatus**

**Child 1**

22. Do you have the MCP immunization card for child 1? 1. Yes 2. No

23 Date of Birth ________________

23.1 Given BCG Vaccine at birth

1. Yes 2. No

23.2 Given 0 dose OPV at birth

1. Yes 2. No

23.3 Given 1^st^ dose DPT/ pentavac at 6 weeks

1. Yes 2. No

23.4 Given OPV 1^st^ dose at 6 weeks

1. Yes 2. No

23.5 Given 2^nd^ dose DPT/ pentavac at 10 weeks

1. yes 2. No 3. NA

23.6 Given OPV 2^nd^ dose at 10 weeks

1. Yes 2. No 3. NA

23.7 Given 3^rd^doseDPT/ pentavac 14 weeks

1. Yes 2. No. 3. NA

23.8 Given OPV 3^rd^ dose at 14 weeks

1. Yes 2.No 3. NA

23.9 Given measles vaccine at 9 months

1. Yes 2. No 3. NA

23.10 Given 1^st^ booster of DPT at 18 months

1. Yes 2.No 3. NA

23.11 Given 1^st^ OPV booster at 18 months

1. Yes 2. No 3. NA

23.12 Given measles booster vaccine at 18-24 months

1. Yes 2. No 3. NA.

23.13 Given 2^nd^ booster DT at 4-5 years

1. Yes 2. No 3. NA

23. 14Given 2^nd^ OPV booster at 4-5 years

1. Yes 2. No . 3. NA

24. Has your child received any vaccination for prevention of pneumonia from thepvt sector?

1. Yes 2. No

25. Did your child receive Vit A drops at any time?

1. Yes 2. No 3. Don’t know 4. Not Applicable *(Note: First dose of Vit A given any time after 6 monthsof age )*

***(skip instruction not in tab)***

26. If yes, approximately how many weeks ago did your child receive the Vit. A

drops?

_____________________________

27. Has your child been de-wormed?

1. Yes 2. No 3. Don’t know 4.NA *(for children below 1 year)*

**5.Nutrition Practices**

32.Was your child breast fed?

1. Yes *(Skip to Q. No.33)* 2. No

32.1 If No. What feed was given to your child*(Code and skip to Q.No. 33)*

1. Cow’s/Buffalo milk/goat milk 2. Formula milk

33. How soon after birth was your child breast fed?

1. Within 1 hour of birth 2. After 1 hour but within 24 hours of birth 3.After 24 hours of

birth

34. For how long was your child exclusively breast fed?

Currently exclusively breast fed

State the number of months

State the number of weeks

Never exclusively breast fed

34.1 State the number of months

34.2 State the number of weeks

35. How long was your child breast fed overall? *(Choose the appropriate answer)*

Continue to breast feed

.. ----Weeks

3. Months ____________

35.1 State the number of weeks

35.2 State thenumber of months

36.At what age (in months) did you start complementary foods?

Specify age ____________

*If not ot started enter 0)*

37. What type of complementary food is being given now?

37.1 Pulses 1.Once every day 2. Not daily 3. Not started 4.Not giving

37. 2 .Cereals 1.Once every day 2. Not daily 3. Not started 4.Not giving

37.3 Vegetables 1. Once every day 2. Not daily 3. Not started 4. Not giving

37.4 Fruits 1. Once every day 2. Not daily 3. Not started 4. Not giving

347.5 Eggs 1. Once every day 2. Not daily 3. Not started 4. Not giving

37.6 Meat 1. Once every day 2. Not daily 3. Not started 4. Not giving

37. 7 Milk other than

breast milk 1. Once every day 2. Not daily 3. Not started 4. Not giving

**6. TheIndexChild***(All questions that follow to be asked only of index child selected from Q. No.8.6. If not an index child thank mother and close interview)*

38.Weight of index child at birth __________________________ (gms)

*(If weight not known enter 00)*

39. Current weight of index child __________________________(gms)

*(If weight not known enter 00)*

39.1. Mid upper arm circumference (MUC) _________________ (cms)

*(If not measured enter as 00)*

1. Red
2. Green
3. Yellow

39.2 mid arm circumference- colour

40. Is index child registered at the ICDS centre?

1. Yes 2. No *(Skip to Q. No. 42)*

41. . Is the child receiving supplementary nutrition from the ICDS centre?

1. Yes 2. No 3. NA*( for children less than 6 months of age)*

42. Was the child *(severe acute malnutrition -SAM)* admitted in the Nutritional Rehabilitation Centre (NRC) during the last 6 months?

1. Yes 2. No 3. NA

**7.Care Seeking Behavior for the Index Child***(this section must be filled only for index children)*

43.Did your child have any of the following signs and symptoms during the illness?

43.1 Fever 1. Yes 2. No 3. Don’t know/remember

43.2 Running nose/Cold/cough 1. Yes 2. No 3. Don’t know/remember

43.3 Fast breathing 1. Yes 2.No 3. Don’t know/remember

43.4 Chest wall in-drawing 1. Yes 2. No 3. Don’t know/remember

43.5 Stridor/Grunt 1. Yes 2.No 3 Don’t know/remember

45. Did your child have measles prior to this illness?

1. Yes 2. No 3. Don’t remember 4. Don’t know

46.Did your child have whooping cough prior to this illness?

1. Yes 2. No 3. Don’t remember 4. Don’t know

47Did you givetreatment for this illness in your child?

1. Yes 2. No*(If no skip to Q. No 78 )*
   1. *48.*What was the first treatment given to the child? Home remedies given 1. Yes 2. No
   2. Child given medicine available at home 1. Yes 2. No
   3. Child given medicine from medical shop 1. Yes 2. No
   4. Child given medicines by ASHA/ANM 1. Yes 2. No

48.5 Took child to a doctor/health facility within

1. 24 hours 1. Yes 2. No *(Skip to 55 )*

49. How many days did you give this treatment?------------

50. What was the status of the child following this treatment?

1. Recovered (*Skip to Q. No. 78*)
2. No change
3. Worsening of symptoms

51.If No change/worsening of symptoms what did you do next?*)*

51.1 .Continued home remedies 1. Yes 2. No

51.2 . Tried different medicine suggested in medical shop 1. Yes 2. No

51.3 Child given medicine by ASHA 1. Yes 2. No

51.4 .Took child to a health facility 1. Yes 2. No*(If Yes Skip to Q No.54.)*

52. What was the status of the child following this treatment?

1. Child recovered *(Skip to Q No 78)* 2 Condition worsened

53. What did you do next?

Specify-----------------------------------------------------

54.Approximately, after how many days following commencement of symptoms *(fever, fast breathing*) did you seek care at a health facility? ________________days

55.Where was the child taken?.(*Circle most appropriate response)*

1. Child taken to the PHC
2. Child taken to CHC
3. Child taken to district hospital
4. Child taken to medical college
5. Child taken to charitable hospital
6. Child taken to private hospital/doctor
7. Child treated by practitioner of Indian medicine- qualified

8. Child treated by traditional healer (unqualified person)

56. .If given Indian medicinespecify if it was:

56.1 Siddha 1. Yes 2. No 3. Don’t know

56.2 Unani 1. Yes 2. No 3. Don’t know

56. 3 Ayurveda 1. Yes 2. No 3. Don’t know

56.4 Homeopathy 1. Yes 2. No 3. Don’t know

57. What was advised for the child?

1. Child given medicinein OP 2. Admitted in the facility*(Skip to 69 )*
2. 3. Referred to another facility*.(Skip to 61 )*

**Details of out-patient treatment**

58.For approximately how many days was this treatment provided?_________________

59.What was the status of the child following treatment?

1. Recovered *(Skip to Q No 76*) 2. Partially recovered and advised to continue treatment till recovery*(Skip to Q No 76 1)* 3. No change4. Condition worsened

60.If No change/condition worsened, what did you do next, specify

--------------------------------------------------------------

61.What was the nature of 2^nd^ treatment given *(Circle most appropriate response)*1. Child taken to the PHC

1. Child taken to CHC (Skip to 63)
2. Child taken to district hospital(Skip to 63)
3. Child taken to medical college(Sk(Skip to 63)ip to 63)
4. Child taken to charitable hospital(Skip to 63)
5. Child taken to private hospital(Skip to 63)
6. Child treated by practitioner of Indian medicine- qualified
7. Child taken to a private doctor for allopathic treatment (Skip to 63)
8. Child treated by traditional healer (unqualified person) (Skip to 63)

*(If option 7 selected ask Q. No.57 else skip to Q. No. 58)*

.62. If given Indian medicinespecify if it was:

62.1 Siddha 1. Yes 2. No 3. Don’t know

62.2 Unani 1. Yes 2. No 3. Don’t know

62.3 Ayurveda 1. Yes 2. No 3. Don’t know

62.4 Homeopathy 1. Yes 2. No 3. Don’t know

63.What was advised?

1. Child given medicine in OP 2. Admitted in the facility*(Skip to Q. No. 69)* 3. Referred to higher Govt. facility*(Skip to Q. No.66 )*

64.How many days was this treatment provided ________________

65. What was the status of your child following **2nd treatment**?

1. Recovered *(Skip to 76)*

2. Partially recovered and advised to continue treatment till recovery*(Skip to 76)*

3. Condition worsened and hospitalized*(Skip to Q. No. 69*

66.Where was the child referred to

1. Child referred to district hospital
2. Child referred to medical college

67.What was advised for the child?

1. Prescribed medicines in OP 2. Admitted in the facility*(Skip to Q. No. 69)*

67. 1.What was the duration of treatment?----------------days

68.What was the status of the child following this treatment?

1. Recovered *(Skip to questions on lab testsQ. No. 76)* 2. Condition worsened and hospitalized *Skip to Q. No69)*

**Details of In-Patient Treatment**

69.In what type of facility was the child hospitalized in?

1. PHC/CHC 2. Govt. District Hospital

3. Govt. Medical College Hospital 4. Pvt. Hospital

5. Charitable/Missionary hospital 6. Others specify______

71.Do you have the discharge notes?

1. Yes 2. No

*(obtain copy of discharge notes if available & write down diagnosis)*

72.Diagnosis___________________________________

73.For how many days was your child hospitalized in this facility?----------------------

74. .What was the status of child following hospital treatment?

1. Recovered *(Skip to section on lab tests Q. No. 71-****Not in tab))*** 2.Partially recovered and continued treatment advised by hospital 3. Was referred to another hospital

**History of Lab.Tests**

76. Were any blood tests or X-rays, scans carried out on your child?

1. Yes 2. No

- - 1. 76. . What tests were carried out?
    2. 76. 1 Blood tests 1. Yes 2. No 3. Don’t know

76.2 X-ray 1. Yes 2. No 3. Don’t know

76.3 Others (specify)--------------- 1. Yes 2. No 3. Don’t know

76.3.1 Specify other lab test

77. Was your child nebulized during this illness?

1. Yes 2. No 3. Don’t remember

77.1. If yes, state number of times the child was nebulized for this illness? _____________

. Don’t remember

78. During your child’s illness did you continue to give the food you usually give to your child?

1.Yes*(Skip to Q. No. 80)* 2. No

79.If No what did you give?

79.1 Breast milk only 1. Yes 2. No 3. NA

79.2 Other Foods (easily digestible food like

porridge, bread, juice, milk 1. Yes 2. No 3. NA

79.3 .Other than breast milk) 1. Yes 2. No 3. NA

80. Did you reduce the number of feeds during the child’s illness?

1. Yes 2. No

**Close interview and thank mother for her cooperation**
